# Practical Notes and Formulæ

**Published:** 1886-12-20

**Authors:** 


### PRACTICAL NOTES AND FORMULÆ.

**Dysentery**—

Vin. ipecacuanhæ, *m.* j, every hour, in water, for dysenteric diarrhœa of children.—*Medical World.*

| | | |
|---|---|---|
| ℞. | Hydrag. perchloridi | gr. j |
| | Ammonii chloridi | gr. j |
| | Tinct. hamamelis | ʒ jss |
| | Aq. dest | Oj |

M. S.—One teaspoonful every hour, when there is much sanious discharge.—*Ibid.*

| | | |
|---|---|---|
| ℞. | Magnes. sulphat | ʒ vj |
| | Acid sulph. arom | ʒ ij |
| | Aq. des | ℥ vijj |

M. S.—One-sixth part every four hours; and pulv. ipecac. co., gr. viij, at bedtime.—*Ibid.*

| | | |
|---|---|---|
| ℞. | Tr. capsici | ʒ ij ss |
| | Tr. ferri perchloridi | ʒ ij ss |
| | Aq. dest., ad | ℥ ij |

M. S.—One teaspoonful in wineglassful of water when craving for liquor occurs.—*Ibid.*

| | | |
|---|---|---|
| ℞. | Chlorat. hydrat | ʒ jss |
| | Potass. bromidi | ʒ ij |
| | Sp. ætheris sulph. co | ʒ ij |
| | Tr. valerianæ | ʒ iij |
| | Aq. dest., ad | ℥ vj |

M. S.—One tablespoonful every two or three hours, in commencing delirium tremens.—*Ibid.*

**Wizard Oil.**—Dr. Douglas (in *Medical World*) writes that the following is a fair analysis of Hamlin's Wizard Oil. The formula dates back to 1866, and hence is not a new discovery. It is a perfect fac-simile:

| | | |
|---|---|---|
| ℞. | Alcohol | 1 pint |
| | Gum camphor | 1 oz. |
| | Oil sassafras | ½ oz. |
| | Tr. myrrh | ½ oz. |
| | Tr. capsicum | ½ oz. |
| | Aqua ammonia | ½ oz. |
| | Chloroform | ½ oz. |

M. S.—I regard this recipe as harmless (and useful too) as Hamlin's famous Wizard Oil, and I believe it is as perfect an analysis as we can get.

